# Body composition-derived BMI cut-offs for overweight and obesity in ethnic Indian and Creole urban children of Mauritius

**DOI:** 10.1017/S0007114519003404

**Published:** 2020-01-06

**Authors:** Harris Ramuth, Sadhna Hunma, Vinaysing Ramessur, Magalutcheemee Ramuth, Cathriona Monnard, Jean-Pierre Montani, Yves Schutz, Noorjehan Joonas, Abdul G. Dulloo

**Affiliations:** 1 Biochemistry Department, Central Health Laboratory, Victoria Hospital, Ministry of Health & Quality of Life, Quatre Bornes, Candos, Mauritius; 2 Department of Endocrinology, Metabolism & Cardiovascular System, Faculty of Science & Medicine, University of Fribourg, Fribourg, Switzerland

**Keywords:** Ethnicity, Adiposity, Asian children, African children

## Abstract

It is increasingly recognised that the use of BMI cut-off points for diagnosing obesity (OB) and proxy measures for body fatness in a given population needs to take into account the potential impact of ethnicity on the BMI–fat % relationship in order to avoid adiposity status misclassification. This relationship was studied here in 377 Mauritian schoolchildren (200 boys and 177 girls, aged 7–13 years) belonging to the two main ethnic groups: Indian (South Asian descent) and Creole (African/Malagasy descent), with body composition assessed using an isotopic ^2^H dilution technique as reference. The results indicate that for the same BMI, Indians have more body fat (and less lean mass) than Creoles among both boys and girls: linear regression analysis revealed significantly higher body fat % by 4–5 units (*P* < 0·001) in Indians than in Creoles across a wide range of BMI (11·6–34·2 kg/m^2^) and body fat % (5–52 %). By applying Deurenberg’s Caucasian-based equation to predict body fat % from WHO-defined BMI thresholds for overweight (OW) and OB, and by recalculating the equivalent BMI values using a Mauritian-specific equation, it is shown that the WHO BMI cut-offs for OB and OW would need to be lowered by 4·6–5·9 units in Indian and 2·0–3·7 units in Creole children in the 7–13-year-old age group. These results have major implications for ethnic-based population research towards improving the early diagnosis of excess adiposity in this multi-ethnic population known to be at high risk for later development of type 2 diabetes and CVD.

The tropical island nation of Mauritius, which is situated in the southern Indian Ocean and east of Madagascar, is the most densely populated area of the African region^(1)^. Since its independence 50 years ago, it has experienced a sustained economic and social development and marked improvement in public health^(2)^ with a current infant mortality rate (14/1000 live birth) and life expectancy (71 and 78 years for males and females, respectively) nearing those observed for upper-middle-income countries^(3,4)^. It has also experienced a surge in CVD morbidity and mortality, largely attributed to an epidemic of type 2 diabetes with a current prevalence of about 21 %^(5,6)^ affecting almost equally its two main ethnic population groups^(7–9)^: Indian (South Asian ancestry) and Creole (Malagasy/African ancestry with European admixture). With the current prevalence of CHD and type 2 diabetes in Mauritius ranked among the highest in the world, the public health and economic burden is huge^(5,6)^. This grim public health situation is bound to deteriorate further since national surveys conducted over the past decades in Mauritius suggest a surge in the prevalence of overweight (OW) and obesity (OB) not only in adults^(5,6,10–12)^ but also in children and adolescents^(13–15)^. While these reflect the trends in the OB epidemic worldwide^(16)^, there is considerable uncertainty as to the actual prevalence of excess fat among individuals given that these prevalence values for OW and OB are defined by international classification systems based upon BMI cut-offs developed on Caucasian-majority populations and therefore may have poor accuracy in the screening of other populations and ethnicities for excess fat^(17–20)^.

Indeed, it has repeatedly been demonstrated that people of Asian origins have higher body fat than Caucasians living in the same country and that Asian children have higher body fatness than would be expected for their BMI compared with Caucasians^(21–24)^. Other studies have shown important differences among children of European Caucasian ancestry, South Indian ancestry and African or Caribbean ancestry^(25,26)^, with Caucasian children having a higher body fat % than children of African or Caribbean descent for a given BMI after controlling for sex and age, but a lower body fat % than South Asian children at the same BMI. In addition, the BMI–fat % relationship also differs among Asian from different origins, with Indians having the highest body fat % and Chinese the lowest for the same BMI when comparing Chinese, Malays and Indians living in Singapore^(27)^. In more recent years, assessment of body composition by the ^2^H dilution technique has shown considerable differences in body composition-derived cut-offs for OB in 8–10-year-old children living in urban areas in China, Philippines, Thailand, Malaysia and Lebanon^(28)^.

Thus, a more comprehensive population-specific and ethnic-specific assessment of the relationship between BMI and body fat % is necessary for an accurate screening of people with excess fat. In this context, a recently reported study in Mauritius, using the ^2^H dilution technique to assess body fat in young adults, has suggested that the predominantly Caucasian WHO-based BMI cut-offs of 25 and 30 kg/m^2^ for OW and OB, respectively, seem valid only for Creole men, but not for Creole women nor for Indian men and women whose BMI cut-offs are 2–4 units lower^(29)^. The present study extends the validation of the WHO BMI cut-offs for excess fat to 7–13-year-old Mauritian children by assessing the relationship between BMI and measured body fat mass (FM) in schoolchildren of this age group according to sex and ethnicity. It compares the measured body fat % in this population sample with that predicted from Caucasian-based equations relating body fat % and BMI in children^(30)^ and generates body composition-derived BMI cut-offs for OB and OW in Mauritian children of both ethnicities in this age range.

## Methods

### Participants

Schoolchildren attending four public primary schools were selected for the study on the basis that the schools were found in an urban region where the physical activity and nutrition transitions have been reported to take place; their location in a catchment area close to the Victoria Hospital (Site of the Obesity Unit, Ministry of Health and Quality of Life). The present study was conducted in conjunction with the consortium on ‘Reducing Obesity Using Nuclear Techniques to Design Interventions in Africa’ Study, whose primary aim was to validate the accuracy of the WHO BMI-for-age as a means of assessing excessive body fatness in a large multi-country African sample of children^(31)^. The recruitment goal of the Reducing Obesity Using Nuclear Techniques to Design Interventions in Africa Study was to enrol at least 150 children in primary schools from each of the twelve study countries. The sexual maturity of children was not taken into account for the following reasons: (i) because of ethnic, religious or cultural sensitivities in several countries against performing objective examination of pubertal stage in children in school class environments and (ii) because international classifications of OW and OB (such as the WHO BMI growth chart) are based on BMI-for-age independently of sexual maturity^(32,33)^. The sampling frame focused on randomly selected samples of schoolchildren from urban areas in an effort to improve comparisons across countries and to examine OB in settings where the physical activity and nutrition transitions have had the greatest impact on childhood OB in Africa and other developing countries^(31)^. In each country, a multi-stage random sampling technique was used to select at least four urban public schools in one district or state followed by school sampling frames of all classes corresponding to the target age group and sex^(31)^. Specifically for Mauritius, the Ministry of Education proposed six schools in the urban and suburban region of Quatre Bornes, covering a catchment area known as zone 4, and subjects were recruited from four of them. The sampling approach aimed at enrolling children of the two main ethnic groups (Indian and Creole) and of both sexes from primary school classes of 4th–6th grades. The classes were chosen at random, and all participants in the selected classes were included in the study; the age of the children across these classes being in the range of 7–13 years. In collaboration with the technical staff and the teachers of these schools, a clinic was set up in the school premises for recruiting participants and collecting data amongst the schoolchildren. A total of 377 children comprising 200 boys and 177 girls were enrolled for the study, namely, 234 Indians (115 boys and 119 girls) and 143 Creoles (eighty-five boys and fifty-eight girls). The sample size was estimated to be sufficient to detect significant sex and ethnic differences in body fat % for the same BMI. Based on the previous data comparing body fat % in adults of these two ethnicities^(29)^, power calculation based upon a statistical power of 80 % and a 5 % significance level indicates that, in order to detect a five-unit difference in body fat % with a population standard deviation of 7 at mean BMI, the required number of subjects should be thirty per subgroup; this calculation was performed using the web software https://clincalc.com/stats/samplesize.aspx. The present study was conducted according to the guidelines laid down in the Declaration of Helsinki, and all procedures involving human subjects were approved by the Ethics Committee of the Ministry of Health and Quality of Life in Mauritius (project protocol: MHC/CT/NETH/RAMU). Written informed consent was obtained from the parents/guardians and verbal assent from the children.

### Anthropometry

Body weight was measured to the nearest 0·1 kg in light clothing and without shoes using a portable SECA^TM^ electronic scale and taking into account the weight of clothes estimated to be about 1 kg. Standing height was measured to the nearest 0·1 cm using a portable stadiometer (Leicester Height Measure) and according to the Standardization Reference Manual of Lohman *et al.*^(34)^. BMI was calculated as the ratio of weight (kg):height-squared (m^2^), and OW and OB were defined on the basis of a sex-specific *z*-score relative to the WHO BMI-for-age reference^(32,33)^, namely those having BMI-for-age *z*-scores >+1sd as OW (including OB) and >+2sd as obese.

### Body composition

In the determination of body composition by the deuterium oxide (D_2_O) dilution technique, a baseline saliva sample (4–5 ml) was obtained from each child for the determination of background isotopic enrichment; the subject chewed a small piece of cotton wool for 2–3 min, and the saliva from the cotton wool was then extracted and collected in a sterile and dry tube. This was followed by the oral administration of a weighed dose (0·5 g/kg body weight) of D_2_O (99·9 % purity, Cambridge Isotope Laboratories). The dose was then washed down by putting 100 ml of locally produced commercial (bottled) drinking water in the dosing container and swallowed through the same straw by the subject. The post-dose saliva samples were collected 3 and 3·5 h after D_2_O ingestion, allowing equilibration with body water compartments. All saliva samples were stored at –20°C until analysis. The ^2^H enrichment in the saliva was measured by a Fourier Transform Infrared Spectrometer using FTIR IR-Affinity (Shimadzu Corporation) in accordance with International Atomic Energy Agency protocol^(35)^. Before saliva measurement, the ^2^H standard was prepared by dilution of D_2_O with bottled water. The infrared spectra were measured in the range 2300–2800/cm. The magnitude of the response obtained from the FTIR was deducted from the ^2^H absorption curve by an algorithm (Isotope.exe software, Shimadzu Corporation). Total body water (TBW) in kg was calculated from the D_2_O dilution space using a correction factor of 4·1 % of ^2^H exchange with the non-aqueous compartment of the body^(35)^. For each child, the average of the 3 and 3·5 h values was used in the final calculation of TBW. Fat-free mass (FFM) was then determined from TBW using Lohman’s age- and sex-specific constant for the hydration of FFM for children^(36)^, FM was calculated as the difference between body weight and FFM, and total fat % was calculated as FM as a percentage of body weight. Due to logistical reasons (time constraints for performing the studies during school days and staff availability), body composition determination using the D_2_O dilution technique could only be performed in 222 children (namely all children in schools 1 and 3 and two-thirds of children in school 4). In 156 of the latter children, body composition was also determined by a single frequency bioimpedance technique using the Tanita BC-418 device often referred to as BIA_8_ (Tanita Corp). The data of these 156 children who had their body composition assessed by both the D_2_O dilution technique and BIA_8_ served as an internal sample for validating the BIA_8_ technique against the reference D_2_O dilution technique, and for the derivation of a regression equation that allows the prediction a D_2_O-equivalent body fat % from that assessed by BIA_8_, namely D_2_O fat % = 2·556 + 1·0176 × BIA_8_ – fat % (*r* 0·85; *P* < 0·001). This equation was used to calculate D_2_O-predicted body fat % in the 155 children (comprising all children in school 2 and one-third of school 4) in whom body composition was assessed only by the BIA_8_ technique. Thus, for the schoolchildren population sample under study (*n* 377), the analysis of data on body composition was performed on pooled data obtained by the D_2_O dilution technique (*n* 222) and by the BIA_8_ technique with the latter values expressed as D_2_O-predicted body fat % (*n* 155). For all data on body composition, the fat mass index (FMI) and FFM index (FFMI) were calculated by dividing FM and FFM by height-squared; the values for FMI and FFMI being expressed as kg/m^2^.

### Data analysis and statistics

Data analyses were performed using statistical software (STATISTIX version 8.0; Analytical Software); the figures and graphics were made using Graphpad Prism Software. All data in the tables are presented as mean values and standard deviations. The analysis of body composition parameters (total fat % or other measures of fat) *v*. anthropometric surrogates of adiposity (BMI or BMI-for-age *z*-score) was performed by linear model procedures including Pearson’s product-moment correlations for determining linear associations between variables and statistical comparisons of the two regression lines for equality of variance, slopes and elevations (i.e. y-intercepts). The analytical software for comparison of regression lines utilises the ANCOVA. It first compares the variances for the two regression lines using Bartlett’s test. Subsequently, assuming homogeneity of variance, it compares the slopes. Assuming homogenous variances and parallel lines, it then tests for differences in the y-intercept. For all tests, significance was set at *P* < 0·05. The accuracy of BMI in the diagnosis of OB was performed using standard diagnostic performance indicators, with excess adiposity defined as percentage body fat above 25 % in boys and above 30 % in girls^(37)^. Using the MEDCALC statistical software version 18.9 (MedCalc Software bvba; http://www.medcalc.org), the standard statistical measures were determined as follows: (i) sensitivity as the proportion of truly obese participants identified correctly as obese by BMI; that is, real or true positive; the remaining being false positive, (ii) specificity as the proportion of truly non-obese participants identified correctly as non-obese by BMI; that is, real or true negatives; the remaining being false negative and (iii) positive and negative predictive values for the total sample, where positive predictive value = true positive/(true positive + false positive) and negative predictive value = true negative/(true negative + false negative). A preliminary analysis of a subset of the data (*n* 153) was published as part of a multi-centre study for validating BMI and comparing childhood OB prevalence in eight African countries^(38)^. However, no analysis was performed and reported in the latter publication pertaining to ethnic differences in BMI–fat % relationship or to body composition-derived cut-offs among Mauritian children according to sex and ethnicity – the main focus of the present study here in a larger cohort of children (*n* 377), and the results reported below.

## Results

### Anthropometry

The mean values and standard deviations for age and anthropometric variables of the schoolchildren are shown in Table 1. With data pooled under ‘All’, boys and girls show no significant differences in any of these parameters. However, when stratified according to ethnicity, and despite no significant differences in mean age (9·7 *v*. 9·9 years, respectively), Indian boys show significantly higher values than Creole boys for body weight (+5·8 kg, *P* < 0·01), height (+3 cm, *P* < 0·01), height-for-age (+ 0·47 *z*-score, *P* < 0·01), as well as for BMI (*P* < 0·05) and BMI-for-age (*P* < 0·01). Among girls, by contrast, no significant between-ethnic differences are observed for height, but Creole girls weighed more than Indian girls (+3·6 kg, *P* = 0·05), and this ethnic difference persisted after normalisation of weight for height or for age as reflected by the significantly higher mean BMI or BMI-for-age *z*-score in Creole girls than in Indian girls (*P* < 0·05).

**Table 1.** Population sample physical characteristics according to sex and ethnicity† (Mean values and standard deviations)

	All				Boys				Girls			
	Boys (*n* 200)		Girls (*n* 177)		Indian (*n* 115)		Creole (*n* 85)		Indian (*n* 119)		Creole (*n* 58)	
	Mean	sd	Mean	sd	Mean	sd	Mean	sd	Mean	sd	Mean	sd
Age (years)	9·9	1·1	9·7	1·0	9·9	1·1	9·8	1·2	9·7	0·9	9·8	1·0
Height (m)	1·39	0·09	1·39	0·09	1·40	0·10	1·37**	0·08	1·39	0·09	1·40	0·09
Height-for-age (*z*-score)	0·26	1·24	0·33	1·2	0·46	1·25	−0·01**	1·18	0·32	1·21	0·36	1·11
Weight (kg)	35·7	13·1	35·4	11·8	38·2	14·3	32·4**	10·6	34·2	11·3	37·8*	12·5
BMI (kg/m^2^)	18·1	4·6	18·0	4·4	18·8	4·9	17·0*	3·9	17·5	4·2	18·9*	4·5
BMI-for-age (*z*-score)	0·22	1·90	0·20	1·62	0·50	2·0	−0·16*	1·78	0·024	1·66	0·58*	1·50

Mean values were significantly different between the two ethnic groups: * *P* < 0·05, ** *P* < 0·01.

† All comparisons were adjusted for age except for the comparison of age.

### WHO BMI categorisation

Using the *z*-score tables of the WHO BMI-for-age growth standards for children^(33)^, the proportions of children in the BMI-for-age categories of underweight, OW including OB (OW+OB) and OB only are shown in Table 2 for the entire population sample studied (*n* 377), according to sex, and also according to ethnicity under each sex; the proportion (%) of children who are only OW or normal weight is shown in Table 2. The results indicate that the proportion of children classified as OB in the entire population is about 19 %, but varies considerably according to both sex and ethnicity. Among boys, this proportion is the highest in Indians (27·8 %) which is more than double that for Creoles (11·8 %), whereas among girls, this proportion is greater in Creoles than in Indians (22·4 *v*. 14·3 %, respectively). The proportion of children with BMI-for-age *z*-scores >+1sd (i.e. OW and OB together) constitutes about 35 % of the whole population sample (37·5 % boys and 31 % in girls) and is also much higher in Indian boys (46 %) than in Creole boys (26 %).

**Table 2.** Proportion (%) of children who are underweight (UW), overweight and obese (OW+OB) or obese only (OB) using BMI-for-age *z*-score cut-offs of <−2sd for UW, >+1sd for OW+OB and >+2sd for OB* (Percentages and 95 % confidence intervals)

	Population		All				Boys				Girls			
	Sample (*n* 377)		Boys (*n* 200)		Girls (*n* 177)		Indian (*n* 115)		Creole (*n* 85)		Indian (*n* 119)		Creole (*n* 58)	
	%	95 % CI	%	95 % CI	%	95 % CI	%	95 % CI	%	95 % CI	%	95 % CI	%	95 % CI
UW	11·7	8·5, 15·0	13·5	8·5, 18·5	9·6	5·0, 14·2	11·3	5·1, 17·5	16·5	8·0, 24·9	13·4	6·9, 20·0	1·7	0·1, 5·9
OW+OB	34·5	29·6, 39·0	37·5	30·5, 44·5	31·1	24·0, 38·2	46·1	36·5, 55·6	25·9	16·0, 35·8	28·6	20·0, 37·1	36·2	23·0, 49·4
OB	18·8	14·8, 22·9	21·0	15·1, 26·9	16·9	10·6, 22·1	27·8	19·2, 36·5	11·8	4·3, 19·2	14·3	6·9, 20·0	22·4	10·8, 34·0
OW	15·7	12·0, 19·3	17·5	12·2, 22·8	14·7	9·5, 19·9	19·1	11·9, 26·3	15·3	7·6, 22·9	15·1	8·7, 21·6	13·8	4·9, 22·7
NW	53·8	48·8, 58·9	48·0	41·1, 54·9	58·8	51·5, 66·0	41·7	32·7, 50·8	56·5	45·9, 67·0	57·1	48·3, 66·0	62·1	49·6, 74·6

NW, normal weight.

* From the data on UW, OW+OB and OB generated by WHO growth chart software, the proportion (%) of children who are overweight (OW) or NW are calculated as follows: NW = those with *z*-scores between −2sd and +1sd = 100 – (UW + (OW + OB)); OW = those with *z*-scores between +1sd and +2sd = (OW + OB) – OB.

### Body composition

**Fig. 1.**
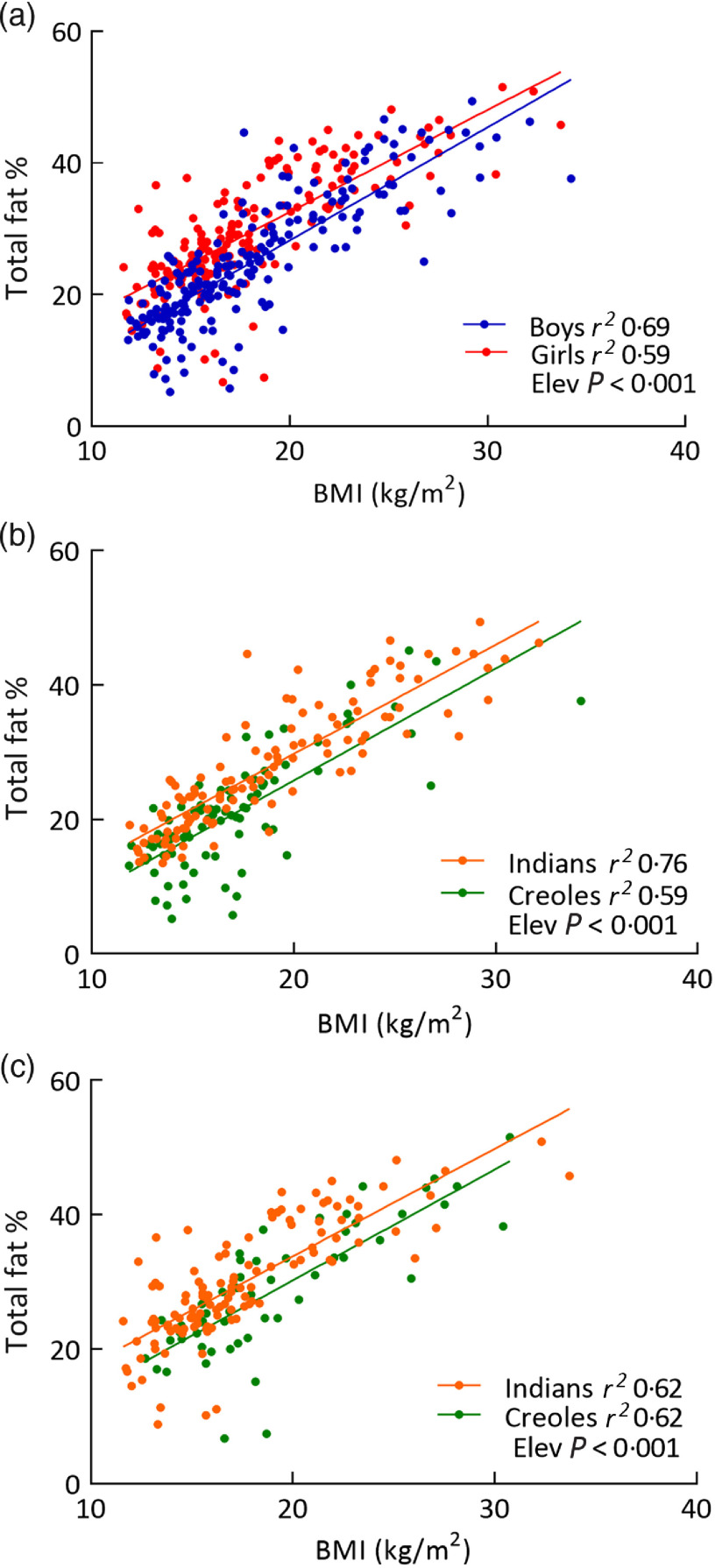
Relationship between percentage body fat (total fat %) and BMI in Mauritian children according to sex (a) and ethnicity in boys (b) and girls (c). The ethnic-specific regression equations are as follows: Indian boys: fat % = −2·64 + 1·62 × BMI; Creole boys: fat % = −7·52 + 1·67 × BMI; Indian girls: fat % = 1·79 + 1·60 × BMI; Creole girls: fat % = −2·71 + 1·65 × BMI.

The mean values and standard deviations for the parameters of body composition are presented in Table 3. The data on four subjects (two boys and two girls) whose body fat % was identified as unphysiological body fat percentage measures (i.e. <5 % body fat) were excluded in this and subsequent analysis. Among the participants, analysis by sex indicates that the measures of adipose mass – that is, total body fat %, FM and FMI – are significantly higher in girls than in boys, while measures of lean mass (FFM and FFMI) are significantly lower in girls than in boys. When analysed according to ethnicity, Indian boys show significantly higher values for all measures of adiposity than Creole boys (*P* < 0·001), but did not differ significantly in the measures of lean mass (FFM and FFMI). Among girls, however, measures of adiposity did not differ significantly between the two ethnic groups, but FFM and FFMI were significantly lower in Indians than in Creoles. However, the application of linear regression models in the analysis of the body composition measures *v*. BMI (which is more sensitive in detecting between-group differences than analysis of means or medians) while confirming the greater adiposity in girls than in boys also reveals that body fat % is higher for the same BMI in Indians than in Creoles both among boys and among girls (Fig. 1). Statistical comparison of the regression lines for boys and girls (Fig. 1(a)) indicates no differences in the slope but significant differences in the elevation or y-intercept (*P* < 0·001) indicating higher body fat % in girls than in boys, and representing a difference in % fat of about five units across the entire BMI range studied (11·6–34·2 kg/m^2^). In boys as well as in girls, comparison of the BMI–fat % regression lines according to ethnicity indicates no significant differences in slope but significant differences in y-intercept (*P* < 0·001) representing a difference in % fat of about 4–5 % units across the BMI range (Fig. 1(b) and (c)). Further regression analysis (data not shown) indicates that similar sex and ethnic differences are observed when body fat % is plotted against BMI-for-age *z*-score and when the analysis is performed on the sub-populations defined as mid-childhood (7–10 years) or late childhood (>10–13 years). They are also observed when the absolute body fat are normalised for height squared, that is, FMI (kg/m^2^), is plotted against BMI or BMI-for-age *z*-score for the entire population sample (age 7–13 years), as well as when the latter analyses are performed on the sub-populations defined as mid-childhood (7–10 years) or late childhood (>10–13 years). Thus, the sex difference as well as ethnic difference with Indian boys and girls showing higher body fat (expressed as fat % or as FMI) for the same BMI or BMI-for-age can be observed in mid-childhood as well as in late childhood.

**Table 3.** Body composition characteristics according to sex and ethnicity† (Mean values and standard deviations)

	All				Boys				Girls			
	Boys (*n* 198)		Girls (*n* 175)		Indian (*n* 115)		Creole (*n* 83)		Indian (*n* 119)		Creole (*n* 56)	
	Mean	sd	Mean	sd	Mean	sd	Mean	sd	Mean	sd	Mean	sd
Body fat (%)	25·0	9·5	29·5***	8·9	27·9	9·2	20·9***	8·7	29·8	8·6	28·8	9·5
FM (kg)	9·96	7·4	11·1**	6·8	11·7	7·9	7·5***	5·8	10·8	6·4	11·8	7·5
FFM (kg)	25·8	6·5	24·3*	6·1	26·4	7·1	25·0	5·7	23·3	5·7	26·3**	6·3
FMI (kg/m^2^)	4·89	3·1	5·6**	3·0	5·65	3·2	3·84***	2·5	5·5	2·9	5·83	3·2
FFMI (kg/m^2^)	13·2	2·0	12·4***	1·9	13·2	2·0	13·2	1·9	12·0	1·7	13·3***	1·9

FM, fat mass; FFM, fat-free mass; FMI, fat mass index; FFMI, fat-free-mass index.

Mean values were significantly different between sex and ethnic groups: * *P* < 0·05, ** *P* < 0·01, *** *P* < 0·001.

† All comparisons were adjusted for age.

### Screening of overfat by BMI v. by body fatness

The data on the proportion of children classified as overfat defined by measured percentage body fat of >25 % for boys and >30 % for girls are shown in Table 4. These values can be compared with overfat defined by anthropometry, that is, using WHO-defined OB as BMI *z*-score > 2sd (OB) or WHO-defined OW and obese as BMI *z*-score > 1sd (OW+OB). For the entire population sample, the proportion of overfat defined by body fatness is shown to be about 42 % which is more than twice the value (18·8 %) obtained by the WHO-defined criterion for OB (i.e. by BMI-for-age *z*-score > 2sd); this discrepancy pertaining to proportion of children undiagnosed as obese by WHO-defined OB being 23 % in the entire population, and varying between 20 and 28 % in all subgroups, except for Creole boys (13·5 % undiagnosed). When the proportion of overfat assessed by body fatness is compared with WHO-defined OW+OB (i.e. by BMI-for-age *z*-score > 1sd), the values are similar in Creole boys (discrepancy close to 0) but still greater in Indian boys as well as in girls of both ethnicities: the percentage undiagnosed as overfat being 8–13 % of each of these three latter sub-groups. Overall, the prevalence of children with measured excess fatness is much higher than that estimated by BMI-based WHO cut-offs for OW and/or OB in all sub-groups (i.e. in both sex and in both ethnicities).

**Table 4.** Proportion (%) of children classified as overfat determined by body fatness (>25 % for boys and >30 % for girls) compared with overfat determined by BMI-for-age *z*-scores as either >+1sd (overweight (OW) + obesity (OB)) or >+2sd (OB) (Numbers and percentages)

	Population	All		Boys		Girls	
	Sample (*n* 373)	Boys (*n* 198)	Girls (*n* 175)	Indian (*n* 115)	Creole (*n* 83)	Indian (*n* 119)	Creole (*n* 56)
Overfat by body fat (%)	42·0	41·9	42·3	53·9	25·3	42·2	44·6
Overfat by BMI (%)							
>+2sd (OB)	18·8	21·0	16·4	27·8	11·8	13·4	22·4
>+1sd (OW+OB)	34·5	37·5	31·1	46·1	25·9	28·6	36·2
Undiagnosed by BMI (%)							
>+2sd (OB)	23·2	20·9	25·9	26·1	13·5	27·8	22·2
>+1sd (OW+OB)	7·5	4·4	11·2	7·8	0·2	12·6	8·4

### Accuracy of BMI in the diagnosis of obesity

The results of the accuracy of BMI in the diagnosis of OB are presented in Table 5. In the entire population sample, the ability of BMI *z*-score > 2sd (WHO-defined OB) to identify the excessively fat children has sensitivity of 38·2 %, specificity of 86·0 %, positive predictive value of 40·9 % and negative predictive value of 84·6 %. The ability of BMI *z*-score > 1sd (WHO-defined OW or obese) had sensitivity of 69·7 %, specificity of 74·4 %, positive predictive value of 40·8 % and negative predictive value of 90·7 %. The results also indicate sex and ethnic dependency in sensitivity, with the lowest sensitivity observed in Indian girls pertaining to the identification of OB (32·6 %) or OW and OB combined (69·4 %).

**Table 5.** Accuracy of BMI in the diagnosis of obesity (OB) (*z*-score > 2sd) as well as overweight (OW) and obesity (*z*-score > 1sd) relative to measured excess fat (>25 % for boys and >30 % for girls) according to ethnicity based on sex* (Numbers and percentages)

	*n*	>2sd cut-off (OB)				>1sd cut-off (OW+OB)			
		Sensitivity (%)	Specificity (%)	PPV (%)	NPV (%)	Sensitivity (%)	Specificity (%)	PPV (%)	NPV (%)
All	373	38·2	86·0	40·9	84·6	69·7	74·4	40·8	90·7
Boys									
Indian	115	51·6	100	100	63·9	82·2	96·2	96·2	82·2
Creole	83	42·8	98·4	90·0	84·0	76·2	90·6	72·7	92·0
Girls									
Indian	119	32·6	100	100	68·0	69·4	100	100	82·3
Creole	56	52·0	100	100	73·3	76·0	93·9	90·5	83·8

* Computations of sensitivity, specificity, positive predictive value (PPV) and negative predictive value (NPV) are described in the section on data analysis and statistics.

### Deviations in body fat % from Caucasian-based BMI predictions

The measured body fat % of Mauritians (Fat % M_M_) can be compared with that predicted from BMI for Caucasians (Fat % C_P_) using predictive equations developed in Dutch children (7–15 years) and validated on several European Caucasian populations by Deurenberg *et al.*^(30)^ and given as:
(1)
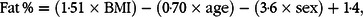

where for sex, males = 1 and females = 0.

**Fig. 2.**
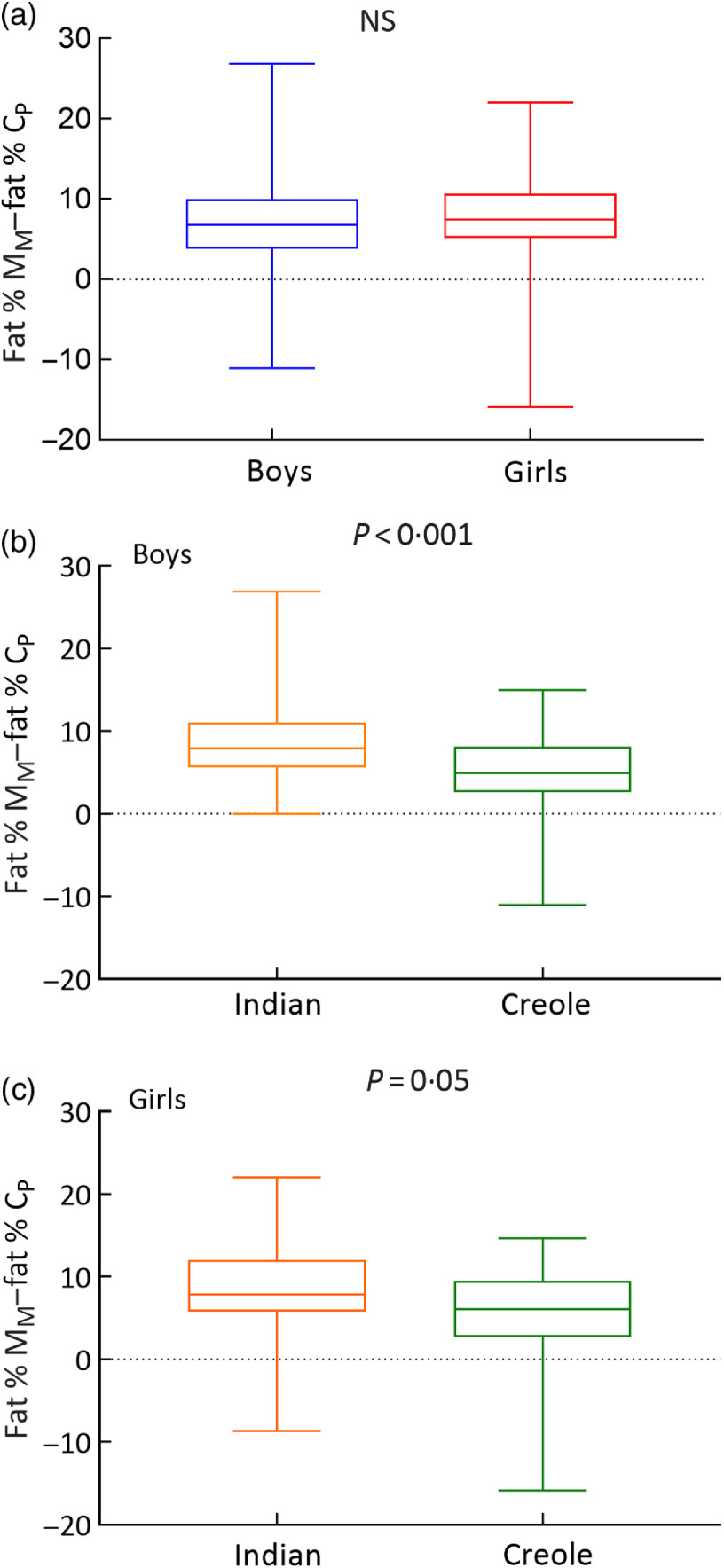
Box and whisker plot showing distribution of values for differences between measured body fat (%) in Mauritians (fat % M_M_) and body fat % predicted from BMI (fat % C_P_) using the Caucasian-based equations of Deurenberg *et al.*^(30)^. The data are presented according to sex (a), ethnicity in boys (b) and ethnicity in girls (c). Each box encloses the data from the second and third quartiles and is bisected by a line at the median value. The tips of the vertical lines indicate the minimum and maximum values.

The distribution of data on the difference between measured fat % *v*. predicted fat % (i.e. fat % M_M_ and fat % C_P_) is presented as box and whisker plots in Fig. 2. Analysis of the data by sex indicates that boys and girls show higher fat % than that predicted for Caucasians by 7–8 % units, while analysis by ethnicity indicates that independently of sex, both Indians and Creoles show higher fat % than that predicted for Caucasians by about 8 % units and 5–6 % units, respectively; the difference between Indians and Creoles also being significant.

### Generation of Mauritian BMI cut-offs for obesity and overweight in children

This is performed in three steps, as described below:

Step 1: Using multivariate regression analysis, a general predictive equation for body fat % from BMI, age, sex and ethnicity can also be developed from the data on the Mauritian children in the 7–13 year age range and is given as:
(2)
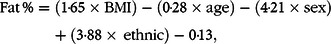

where for sex, males = 1 and females = 0; and for Ethnic, Indians = 1 and Creoles = 0.

Using this predictive equation valid for children aged 7–13 years, body fat % can be determined from their BMI, sex, age and ethnic group.

Step 2: From the above Deurenberg’s equation predicting body fat % from BMI in Caucasian children (Equation 1), the predicted body fat % that corresponds to WHO BMI cut-offs for obese and OW in the age range of 7–13 years is calculated and presented according to ethnicity and sex in Table 6.

**Table 6.** Comparison of BMI cut-off points for obesity and overweight proposed by the WHO using Caucasian data (http://www.who.int/growthref/) with calculated BMI equivalents for Mauritian Indian and Creole boys and girls derived from regression equations predicting body fat % from BMI, age, sex and ethnicity

BMI cut-offs for obesity					BMI cut-offs for overweight				
	Caucasian		Mauritian equivalent			Caucasian		Mauritian equivalent	
Age (years)	BMI (kg/m^2^)	Fat %	Indian	Creole	Age (years)	BMI (kg/m^2^)	Fat %	Indian	Creole
Boys					Boys				
7	19·0	21·6	15·0	17·5	7	17·0	18·6	13·1	15·6
8	19·7	21·9	15·1	17·7	8	17·4	18·5	13·0	15·6
9	20·5	22·5	15·5	18·0	9	17·9	18·5	13·0	15·6
10	21·4	23·1	15·9	18·4	10	18·5	18·7	13·2	15·7
11	22·5	24·1	16·5	19·0	11	19·2	19·1	13·4	15·9
12	23·6	25·0	17·0	19·5	12	19·9	19·4	13·6	16·1
13	24·8	26·1	17·7	20·2	13	20·8	20·1	14·0	16·5
Girls					Girls				
7	19·8	26·4	15·4	17·7	7	17·3	22·6	13·0	15·3
8	20·6	26·9	15·7	18·0	8	17·7	22·5	12·9	15·3
9	21·5	27·6	16·1	18·4	9	18·3	22·7	13·1	15·4
10	22·6	28·5	16·7	18·9	10	19·0	23·1	13·3	15·6
11	23·7	29·5	17·3	19·5	11	19·9	23·7	13·7	16·0
12	25·0	30·7	18·1	20·3	12	20·8	24·4	14·1	16·4
13	26·2	31·9	18·8	21·0	13	21·8	25·2	14·6	16·9

Step 3: For a given age, the predicted body fat % (corresponding to Caucasian BMI cut-offs) is then used to recalculate the BMI for Mauritians using the ethnic-specific prediction (Equation 2) in order to obtain a BMI level for Mauritian children that is equivalent with the WHO cut-offs for OB and OW. The derived BMI cut-offs for each ethnic group (Indians and Creoles) according to age and sex are shown in Table 6. It is shown that for a given sex, age and ethnicity, the BMI of Mauritian children who had the same body fat % as Caucasians is 2–8 units of BMI lower than Caucasians. For example, at 10 years of age, Indian and Creole boys with a BMI of 15·9 and 18·4 kg/m^2^, respectively, have the same level of body fat % (23·1 %) as Caucasians with a BMI of 21·4 kg/m^2^. Among 10-year-old girls, Indians and Creoles with BMI of 16·7 and 18·9 kg/m^2^, respectively, have the same level of body fat % (28·5 %) as Caucasians with a BMI of 22·6 kg/m^2^.

## Discussion

The present study conducted in Mauritian schoolchildren, encompassing a wide range of BMI from 11·6 to 34·2 kg/m^2^ and body fat % from 5 to 52 %, reveals important ethnic differences in body composition during childhood; for the same BMI, age and sex, Mauritian children have more body fat % than that predicted for Caucasians, and among the Mauritian children, those of South Asian descent (the Indians) have more body fat and less lean mass than those of African-Malagasy descent (the Creoles).

### Prevalence of excess adiposity

Based on the WHO growth charts of BMI-for-age^(33)^, the proportion of these children classified as OW or obese is 35 %. This high value reflects the on-going surge in childhood OW and OB on the island since the turn of this century as documented by two National Nutrition Surveys conducted in 2004 and 2012^(13–15)^ indicating that the prevalence of OW and OB in children aged 5–11 years increased from 15 to 22 % within less than a decade. These prevalence values are, however, likely to be underestimated based on our findings here of major discrepancies in the prevalence based on WHO-based definitions of OW and OB and that based on measured body fat. Using cut-offs for excess body fat defined as >25 % for boys and >30 % for girls^(37)^, the prevalence of children with excessive body fat is found to be more than double that determined by the BMI-for-age definition of OB (42 *v*. 18·8 %). This marked underestimation of OB defined by BMI criterion as opposed to measured excessive fat is found both in boys (41·9 *v*. 21 %) and in girls (42·3 *v*. 16·4 %), although the extent of this underestimation varies considerably according to ethnicity among girls. Indeed, while the prevalence of excessive fatness is about two times higher than WHO-defined OB among boys of Indian and Creole ethnicities (53·9 *v*. 27·8 %; 25·3 *v*. 11·8 %, respectively) as well as in Creole girls (44·6 *v*. 22·4 %), it is over three times higher among Indian girls (42·2 *v*. 13·4 %).

### Accuracy of BMI cut-offs

In fact, in our evaluation of the accuracy of BMI in the diagnosis of OB using cross-tabulation, the sensitivity of WHO-defined BMI cut-offs for OB in identifying boys with a body fat % over 25 % and girls with a body fat % over 30 % is ethnicity-dependent. Although the sensitivity is low (about 50 % or less) and specificity is high (>98 %) in all four subgroups according to ethnicity nested under sex, the lowest sensitivity (32·6 %) is observed in Indian girls such that only one in three Indian girls with a body fat % above 30 % is identified as obese by the WHO-based BMI classification. Overall, depending on sex and ethnicity, the sensitivity analysis suggests that only between 33 and 52 % of the children with body fat in excess of 25 % in boys and 30 % in girls are identified as obese by the WHO BMI-for-age cut-offs. Compared with the sensitivity value of 38·2 % reported here for the entire population of children (*n* 373; aged 7–13 years), a similarly low sensitivity value of 41 % was also found in the diagnosis of OB by BMI in a subset (*n* 153) of these Mauritian children in the tighter age range of 9–11 years^(38)^. Low sensitivity values in the range of about 10–60 % for BMI-defined OB have also been reported in recent studies applying the ^2^H dilution method to measure body fat in 8–11-year-old children living in urban areas of several low- and moderate-income countries in Asia^(28)^ and Africa^(38)^. These findings therefore underscore the need for developing countries to generate their own age-specific, sex-specific and ethnic-specific BMI classification.

### Generating Mauritian-specific BMI cut-offs for overweight and obesity

Indeed, by applying the approach of using the Caucasian equation developed and validated on Europeans^(30)^ to predict body fat % from WHO-defined BMI thresholds for OW and OB, and the recalculation of equivalent BMI values using ethnic-specific equations, we are able to derive Mauritian BMI cut-offs for OB and OW in children of each ethnic group (Indians and Creoles) according to sex (Table 6). In line with the study of Liu *et al.*^(28)^ reporting lower body composition derived cut-offs for OB in 8–10-year-old children living in urban areas in several Asian countries (China, Philippines, Thailand Malaysia and Lebanon), we found here that in Mauritian children of similar age group (i.e. 8–10-year-old children), the WHO BMI thresholds for OB would need to be lowered by as much as 4·5–6 units in Indians and 2–4 units in Creoles (Table 7). It is noticeable that among all these ‘Asian’ populations, Mauritian Indian boys and girls have the lowest BMI cut-offs, which contrast with Mauritian Creole boys and girls who have the highest BMI cut-offs, the between-ethnic difference in threshold lowering being a reflection of the differences in their ethnic specific BMI–fat % relationships.

**Table 7.** Comparison of BMI cut-off points for obesity proposed by the WHO using Caucasian data with calculated BMI equivalents for Mauritian Indian and Creole (data from Table 6 here) and compared with the data reported by Liu *et al.*^(28)^ for Chinese, Lebanese, Malay, Filipino and Thai*

	Boys			Girls		
Age (years)…	8	9	10	8	9	10
Caucasian						
BMI (kg/m^2^)	19·7	20·5	21·4	20·6	21·5	22·6
Body fat %	21·9	22·5	23·1	26·9	27·6	28·5
Predicted BMI equivalent (kg/m^2^)						
From Liu *et al.*^(28)^						
Chinese	16·4	16·6	16·9	17·2	17·5	17·9
Lebanese	16·8	17·0	17·2	17·0	17·3	17·7
Malay	15·8	16·0	16·3	16·5	16·8	17·3
Filipino	16·6	16·9	17·3	16·4	16·8	17·4
Thai	15·7	16·0	16·3	15·8	16·1	16·5
Mauritian (present study)						
Indian	15·1	15·5	15·9	15·7	16·1	16·7
Creole	17·7	18·0	18·4	18·0	18·4	18·9
Deviation from WHO cut-offs						
Mauritian Indian	4·6	5·0	5·5	4·9	5·4	5·9
Mauritian Creole	2·0	2·5	3·0	2·6	3·1	3·7

* Note that all the BMI equivalents are derived from regression equations for predicting body fat % from BMI assessed in each country where body composition was determined by the D_2_O dilution technique as the reference method.

### Sex and ethnic differences in BMI–fat relationships

Indeed, the application of linear regression analysis, which provides a more sensitive approach than comparison of means or median to detect differences in body fat % for the same BMI, reinforces the existence of these sex and ethnic differences in the BMI–fat % relationship and indicates that they occur across the entire range of BMI or BMI-for-age; that is, these differences encompass all BMI categories (underweight, normal weight, OW and obese) with Indians showing higher body fat % (by 4–5 % unit) than Creoles among both boys and girls. Furthermore, using FMI – which is independent of the FFM component of body weight – in comparing the relationships between fatness and BMI, the observed differences due to sex and ethnicity are also present when FMI is regressed against BMI. The robustness of these sex and ethnic differences is further underscored by their persistence when performing a sensitivity analysis with the omission of subjects with extreme values (very low or very high) BMI-for-age (*z*-scores <–3sd and >+3sd), as well as when the analyses are performed on the sub-populations defined as mid-childhood (7–10 years) or late childhood (>10–13 years). Thus, the sex difference, as well as ethnic difference, with Indian boys and girls showing higher body fat (expressed as fat % or as FMI) for the same BMI or BMI-for-age can be observed in both mid-childhood and in late-childhood.

### Ethnic differences in body composition: during growth and in adulthood

With regard to the present study showing that Mauritian children are fatter than Caucasians for the same BMI, and that within the island, Indian children have more body fat and less FFM than Creole children for the same BMI, age and sex, the relative importance of genetics and environment in these differences needs to be studied. Nonetheless, it is of interest to note that the sex and ethnic differences in body composition in this population aged 7–13 years are also observed when the analysis is performed on the sub-populations defined as mid-childhood (7–10 years) or late childhood (>10–13 years). These data indicate that ethnic differences, with Indians showing higher fat % or higher FMI (and lower FFMI) than Creoles for the same BMI-for-age, can be observed in both age group categories of mid-childhood and late childhood. While the stages of puberty were not examined in our study, the occurrence of ethnic differences in body composition during mid-childhood raises the possibility that they may already be present prior to puberty. Such a notion, which would underscore a genetic basis for the ethnic differences in body composition, warrants investigations in early childhood. In this context, it is also of interest to compare these ethnic differences in anthropometry and body fat % described here in children and those previously reported in adults from a previous study conducted in Mauritius that also used the ^2^H dilution technique as reference for assessing body composition^(29)^. Although sex differences in the relationships between total body fat % and BMI observed here in children are also found in young adults (with values for females greater than those for males), the ethnic differences in these relationships in adults are, however, sex-specific. Indeed, unlike in both boys and girls where Indians show greater body fat % for the same BMI than Creoles, these ethnic differences are, however, observed only in men but not in women – among whom both ethnicities were found to be equally fatter than Caucasian women of the same BMI. The presence of ethnic differences between Indian and Creole in both sexes during childhood but which persist only in males in adulthood is intriguing. Among potential explanations are (i) a high degree of genetic admixture of Indian with African/Malagasy ancestry among Creole women, (ii) hormonal changes which occur during puberty and (iii) changes in diet and/or physical activity behaviours, perhaps related to changes in weight control perceptions, later in adolescence and adulthood.

### Study limitations

First, although the study was conducted on a relatively large sample of children (*n* 377) from schools with classes corresponding to the target age-group, sex and ethnicity, all the four schools were in one urban district such that the children may not be representative of the general population. While the reason for choosing an urban region was to assess excess adiposity in childhood in areas where the physical activity and nutrition transitions have had probably the greatest impact, it should also be pointed out that in Mauritius, which is a very small island state with a high population density of 625/km^2(39)^, the distinction between urban and rural is narrow. Nevertheless, the relatively large sample size allowed analyses to be performed based on sex and ethnicity (the two main ethnic groups which make up >95 % of the Mauritian population). Additionally, meaningful investigations of the BMI–fat % relationship were possible as a result of the large range of BMI (11·6–34·2 kg/m^2^) and body fat % (5–52 %) among the study population. Second, the sexual maturity of children was not assessed, and hence variations in pubertal stages may contribute to the observed differences in sex and ethnic differences in the BMI–fat % relationship. However, the main aim of the study was to validate the WHO BMI classification for excess adiposity, which is not based upon pubertal stage but upon BMI-for-age^(32,33)^. Indeed, in both public health research and in clinical practice, both BMI and body fat reference percentiles for children and adolescents are developed as age- and sex-specific rather than based on sexual maturity and Tanner stages^(32,33,40–43)^. In addition, the conversion of measures of TBW (as assessed by D_2_O or BIA techniques) to total FFM and body fat utilises established age- and sex-specific constants for the hydration of FFM and is not based on sexual maturity^(36)^. It is also not known whether puberty ratings vary in accuracy across ethnic groups. However, it is a reasonable assumption that in children below the age of 10 years, particularly in boys, the vast majority would be in the pre-pubertal phase independently of ethnicity^(43)^. Thus, in the younger age subgroup defined as mid-childhood (7–10 years old), we believe that sexual maturity is unlikely to be an important factor in the interpretation of our findings of ethnic differences with Indians showing higher body fat (expressed as fat % or as FMI) for the same BMI or BMI-for-age. Nonetheless, division of the sub-groups based on sexual maturity rather than age only may be more informative in future studies, although ethical issues may preclude this test of pubertal stages. Third, differences in methodology for measuring body fat may introduce errors in our calculations of differences between fat % measured in our Mauritian population sample using the D_2_O dilution technique as reference and that predicted from BMI using the Deurenberg equation derived by hydrodensitometry^(30)^. Indeed, the TBW measured in our study is used to determine FFM which is then subtracted from body weight to obtain body fat; this series of calculations thereby increases the potential for ‘propagation errors’. However, a mean bias of 0·6 % was found when body fat % was assessed by these two methods in Caucasians, which indicates a close agreement^(44)^. Moreover, a small bias (at about 1 % or less) was reported in body fat % assessed using D_2_0 dilution *v*. the 4-C model in both Caucasians^(44)^ and Asians^(45)^. Fourth, although the method for assessing TBW and predicting body fat % based on the two compartment model is valid, this method assumes a constant hydration factor in children of a given age. The limitation of this assumption is that FFM hydration can vary according to pubertal stage and ethnicity, which may lead to erroneous estimations of FFM, thus contributing to population subgroup differences in FFM and FM. However, as Deurenberg & Deurenberg-Yap^(46)^ point out in their review about the hydration of FFM, the majority of studies have failed to find significant ethnic differences (black *v*. white Americans, Caucasians *v*. Asians (Chinese, Malays and Indians)); any differences that were identified were deemed too small to be of biological relevance. Finally, cut-offs of body fat as 25 and 30 % for classifying boys and girls, respectively, as overfat are used independently of age and hence raise the question of whether age-specific cut-off values may not be more appropriate. However, these latter cut-offs were derived by Williams *et al.*^(37)^ from the relationship between body fatness and cardio-metabolic risk in childhood, and a marked increase in cardiometabolic risk profile (cholesterol, lipoprotein ratios, blood pressure) at body fat exceeding 25 % in boys and 30 % in girls has been shown across a wide age range (5–18 years), that is, independently of age among children and adolescents.

### Conclusion

The present study underscores the substantial extent to which international BMI cut-offs for OW and OB underestimate the scale of the prevalence of excessive fatness in Mauritian children, particularly in those of Indian ancestry: the prevalence of those with excessive body fat is found to be more than double that determined by the BMI-for-age definition of OB. Furthermore, the analysis of body composition reveals important ethnic differences in adiposity: for the same BMI, age and sex, Mauritian children have more body fat % or higher FMI than predicted for Caucasians, and among Mauritian children, Indians have more fat (and less lean mass) than Creoles. Further studies are required to cross-validate the multivariate equation for predicting body fat % from BMI, age, sex and ethnicity, and the body composition derived cut-offs for OB and OW according to sex and ethnicity in the children in this age group, and also in earlier childhood. Such studies have major public health implications for ethnic-based population research towards improving the early diagnosis of excess fatness in this multi-ethnic population known to be at high risk for later development of type 2 diabetes and CVD. It also has implications for research towards a better understanding of their underlying causes, as well as for monitoring the efficacy of intervention studies geared at developing better strategies to prevent, hinder or curtail the development of OB and co-morbidities in this, as well as other, ‘at risk’ populations worldwide.
